# The Correct Indication to Induce Labour in a Swiss Cantonal Hospital

**DOI:** 10.3390/jcm12206515

**Published:** 2023-10-14

**Authors:** Munachimso Kizito Mbata, Maria Boesing, Giorgia Lüthi-Corridori, Fabienne Jaun, Grit Vetter, Jeanette Gröbli-Stäheli, Anne B. Leuppi-Taegtmeyer, Brigitte Frey Tirri, Jörg D. Leuppi

**Affiliations:** 1 University Institute of Internal Medicine, Cantonal Hospital Baselland, Rheinstrasse 26, 4410 Liestal, Switzerland; 2 Faculty of Medicine, University of Basel, Klingelbergstrasse 61, 4056 Basel, Switzerland; 3 Department of Gynaecology and Obstetrics, Cantonal Hospital Baselland, Rheinstrasse 26, 4410 Liestal, Switzerland; 4 Hospital Pharmacy, Cantonal Hospital Baselland, Rheinstrasse 26, 4410 Liestal, Switzerland; 5 Department of Patient Safety, Medical Directorate, University Hospital Basel, Schanzenstrasse 55, 4056 Basel, Switzerland

**Keywords:** induction of labour, labour outcome, Bishop score, maternal outcome, neonatal outcome, success of labour, audit, guidelines

## Abstract

Background: Induction of labour (IOL) is a way to stimulate the onset of labour using mechanical and pharmacological methods. IOL is one of the most frequently performed obstetric procedures worldwide. We aimed to determine compliance with guidelines and to investigate factors associated with the success of labour. Methods: In this retrospective, observational study, we analysed all induced deliveries in a Swiss hospital between January 2020 and December 2022. Results: Out of 1705 deliveries, 349 women underwent IOL, and 278 were included in this study, with an average age of 32 years (range 19–44 years). Most of the women were induced for missed deadlines (20.1%), the premature rupture of membranes (16.5%), and gestational diabetes mellitus (9.3%), and there was a good adherence to the guideline, especially with the indication and IOL monitoring (100%). However, an improvement needs to be made in measuring and documenting the Bishop score (41%). The success of labour was associated with multiparity (81.8% vs. 62.4% *p* = 0.001) and maternal non-obesity (73.4 vs. 54.1% *p* = 0.026). Conclusions: An improvement is needed in the measurement and documentation of the Bishop score. Further research is needed to confirm the found associations between parity, obesity, and the success of IOL.

## 1. Introduction

Induction of labour (IOL) is a way to stimulate the onset of labour using artificial methods [1]. The rate of IOL has shown a consistent increase on a global scale, although it can vary from country to country. This upward trend can be attributed to multiple factors, including pre-existing medical conditions, maternal age, parity, body mass index (BMI), and foetal weight [2,3,4,5]. IOL is advised to be carried out only when the benefits outweigh the risks [6]. It is typically carried out by administering oxytocin or prostaglandins to the expectant mother or through the artificial rupture of the amniotic membranes. Despite its standard practice, it is essential to recognise that labour induction is not entirely devoid of risks, and expecting women may find it uncomfortable [7]. Different indications for inducing labour include the premature rupture of the amniotic membrane, post-term pregnancy, diabetes, and severe foetal growth restriction. IOL aims to prevent adverse maternal and foetal outcomes [2,8]. However, induction should be avoided in cases where there is an abnormal foetal presentation, placenta previa, umbilical cord prolapse, or active genital herpes infection [9,10].

To determine if the cervix is favourable or not and to assess if there will be a need for cervical ripening, the Bishop score is always recommended [11]. The Bishop scoring system assesses multiple parameters such as cervical dilation, position, effacement, consistency, and foetal station. Cervical dilation, effacement, and foetal position are assessed using a scoring range of 0 to 3 points, whereas cervical position and consistency are assigned scores ranging from 0 to 2 points [11,12,13].

The timing of labour induction is a crucial consideration, warranting careful evaluation and implementation only when medically justified. Non-medically indicated IOL should be avoided until term pregnancy, as studies have indicated higher rates of neonatal morbidity and mortality in early term deliveries compared to term deliveries [14,15]. On the other hand, prolonged pregnancy, defined as gestation beyond 42 weeks, is associated with an increased risk of perinatal complications and the likelihood of requiring instrumental birth or caesarean delivery [16].

With IOL being the process of stimulating the onset of uterine contractions, various methods can be used, such as pharmacological methods, which include the use of oxytocin, prostaglandin E1 (PGE1), and prostaglandin E2 (PGE2). Of these, some studies have shown that PGE1 lowered the rates of caesarean sections and shortened the period from induction to vaginal delivery, whilst PGE2 was shown to be safer due to its lower risk of uterine hyperstimulation and tachysystole [17,18,19]. The mechanical method of inducing labour includes the use of balloon catheters and amniotomy. Induction is shown to continue with oxytocin after the catheter has been removed, which explains the higher use of oxytocin compared to prostaglandins [20,21]. Amniotomy should only be performed when indicated and if the membranes are reachable, thus eliminating the necessity for pharmacological interventions [22,23,24].

We audited the correct indication and methods of induction at the Cantonal Hospital Baselland in Switzerland, alongside factors that could affect the outcome of labour and adherence to the guidelines. Depending on the findings and the resulting outcomes, factors that can influence the labour outcome differ according to studies [25,26,27].

As IOL is a frequently performed obstetric procedure, there may be more factors associated with the success of induction; future studies can investigate these factors further.

The main aim of this study was to evaluate the appropriateness of labour induction in the Department of Gynaecology and Obstetrics at the Cantonal Hospital Baselland (KSBL). We looked at indications, measures taken, and methods of induction. We also compared the cases of induction with clinical guidelines generated by the German Society of Gynaecology and Obstetrics (DGGG) in cooperation with the Austrian Society of Gynaecology and Obstetrics (OEGGG) and the Swiss Society of Gynaecology and Obstetrics (SGGG) [20] in addition to KSBL internal guidelines, while simultaneously assessing adherence to the recommended protocols.

The secondary objectives were to evaluate the association between maternal age and the success rate of labour induction as well as rates of caesarean delivery.

## 2. Materials and Methods

### 2.1. Study Design and Setting

We performed a retrospective, observational, single-centre study at the Cantonal Hospital Baselland (KSBL) in the Department of Gynaecology and Obstetrics in Switzerland. 

### 2.2. Study Outcomes

The primary outcome was the adherence to the official guideline generated by the DGGG in cooperation with the OEGGG and the SGGG and KSBL internal guidelines.

Secondary outcomes included the evaluation of the success of IOL and maternal and neonatal outcomes. The success of IOL was defined as delivery achieved through vaginal delivery. Unsuccessful IOL was defined as the inability to achieve a vaginal delivery, meaning that a caesarean section (CS) was the mode of delivery [28,29]. Unsuccessful IOL has not been given a clear global definition, as it is defined differently across different studies [30,31].

### 2.3. Patient Population: Inclusion and Exclusion Criteria

**Figure 1:**
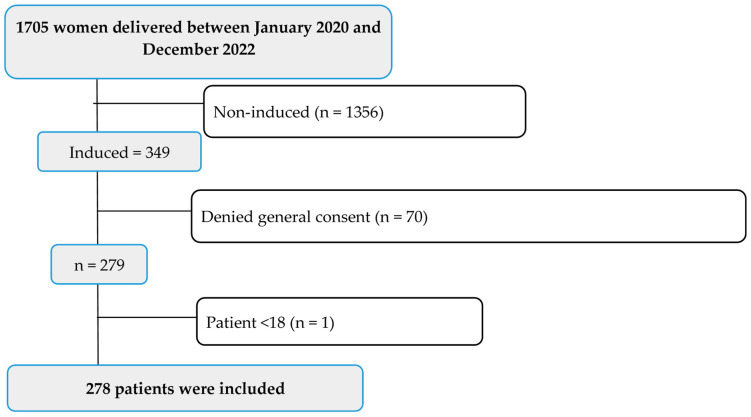
Flowchart of the patient selection and enrolment process.

The study population included all induced deliveries in the Cantonal Hospital Baselland between January 2020 and December 2022. Patients who denied general consent for their health-related data and samples to be used for research purposes were excluded from this study. Inclusion criteria were defined as the following: Patients aged 18 years or older and if the patient was pharmacologically or mechanically induced. The exclusion criteria were as follows: patients who had declined general consent for the use of health-related data and samples, patients younger than 18 years, and non-induced labour (Figure 1).

### 2.4. Data Collection Process

The controlling department compiled a list of patients for 2020–2022 using the Swiss Diagnosis Related Groups (SDRG) code and the childbirth procedure code. A list was provided by the Gynaecology and Obstetrics department of the KSBL with the number of deliveries and inductions per year. The IT department also provided a list of patients who had given or denied their general consent. The lists mentioned above were merged, and the resulting list was then imported into a research electronic data capture (RedCap database) [32,33], which consisted of specific electronic case report forms (eCRF) for the purposes of data capture. Patients’ clinical routine data were manually extracted and entered into the eCRF from the electronic patient files in “Polypoint KIS” (Polypoint AG, Gümlingen, Switzerland) and ‘’ Viewpoint 5 and 6” (GE HealthCare, Munich, Germany).

### 2.5. Statistical Analyses

Statistical analyses were performed using R version 4.3 (R Foundation for Statistical Computing), Statistical Package for the Social Sciences software (SPSS), Version 24 (IBM), RedCap, version 13.8.1, and Microsoft Excel, version 16.0.5278.1000. Categorical variables were shown in absolute and relative frequencies. Continuous variables were shown as mean +/− standard deviation (SD) when normally distributed or median and interquartile range (IQR) when not normally distributed. Normal distribution was verified using histograms and Q-Q plots.

Categorical variables were analysed with the Chi-squared test or Fisher’s exact test. For hypothesis testing, we considered *p*-values < 0.05 to be statistically significant.

## 3. Results

### 3.1. Patient-Based Baseline Characteristics

The study population included 278 induced deliveries. Out of 1705 total deliveries, 278 were included, whilst 1427 were excluded for several reasons (Figure 1). The baseline characteristics of the patients are shown in Table 1. The overall mean age was 32.18 years, and the median length of the hospital stay (LOS) was 5 days. Maternal age ranged from 19 to 44 years old; more than one-fourth of the study population were aged between 35 and 44 (n = 80, 28.8%). Some women had at least one pre-existing disease (n = 77, 27.7%), with iron deficiency anaemia being the most frequent (n = 31, 11.1%). The median gestational age (GA) in weeks at delivery was 40.4 (IQR = 39.4–41.3) (Table 1).

### 3.2. Implementation of the Guidelines in the Case of an Indication of IOL

Table 2 provides information on the correct indication of IOL in KSBL following the official guideline. The notable occurrences of missed deadlines 20.1%, PROM 16.5%, and GDM 9.3%, which accounted for most of the single reasons for induction was observed in most cases. Induction was carried out in women with oligohydramnios, polyhydramnios, intrahepatic cholestasis, and macrosomia representing 1.1%, 1.1%, 1.8%, and 3.2%, respectively. No inductions were recorded for HELLP syndrome. IUGR/SGA and preeclampsia accounted for 3.2% for each. In 1.4% of the cases, the indication for IOL was not recorded. Induction without a medical reason was noted in 1.1% of the deliveries, whilst 38% of inductions were attributed to multiple indications (Table 2).

### 3.3. Implementation of Guidelines Based on Other Recommendations

Before each IOL, all pregnant women were informed about the procedure and what it involved, which was documented via written consent. The Bishop score was calculated and documented in 41% of the cases, all of which were unfavourable. Out of the induced cases, none of the women received amniotomy alone. However, 11.8% of the women received administration of only oxytocin, which deviates from the guideline recommendation. The balloon catheter (single and double) in combination with oxytocin was used in 3.6%. The balloon catheter combined with prostaglandin E1 (misoprostol) was not used, neither was its combination with prostaglandin E2 (propess) in the analysed cases. One hundred and two women (36.7%) received PGE2 alone; as PGE2 is not limited to sole administration, some women received it alongside PGE1. The guideline recommended that multiple methods of IOL could be used during the induction procedure, as some pregnant women can have multiple indications for IOL and as the cervix takes time to mature. Cardiotocography (CTG) was conducted 30 min before, during, and after the IOL in 100% of the observed cases until childbirth. Pathological CTG was observed in 12% of the cases (Table 3).

### 3.4. Factors and Outcome of Successful IOL

The proportion of mothers that experienced unsuccessful induction was 29.1%, and among those cases, 29.6% were observed in mothers older than 35 years. Factors associated with successful IOL were multiparity (81.8% vs. 62.4% *p* = 0.001) and maternal non-obesity (73.4 vs. 54.1% *p* = 0.026) (Table 4).

### 3.5. Maternal and Neonatal Outcomes

**Figure 2:**
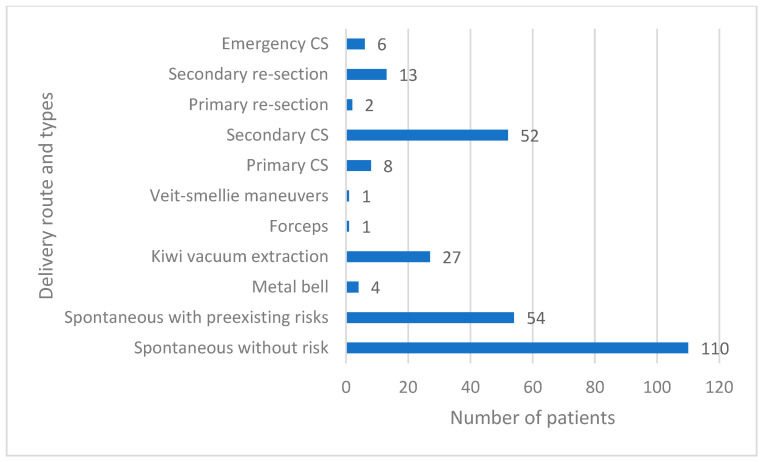
Types of vaginal and caesarean deliveries. CS—caesarean section.

Regarding the delivery route, vaginal birth emerged as the most common (70.9%). Birth injuries were observed in 54.7%, and these included episiotomy (11.2%), vaginal (26.6%) and perineal tear (28.8), labia (15.8%) and para-urethral tear (0.7%), as well as cervical tear (0.4%) (Table 5). Fifty-six percent of the vaginal deliveries were spontaneous without any risks, whilst 27% had some maternal or neonatal risks, and the rest were instrumental deliveries, which was mostly indicated by foetal distress and malpresentation as well as maternal exhaustion. Out of the deliveries by CS, 9.9% had a planned CS, 64.2% underwent an unplanned CS, 18.5% of the cases involved women that had previously given birth through planned and unplanned CS, and 7.4% had an emergency delivery (Figure 2).

## 4. Discussion

To the best of our knowledge, our study is the first one in Switzerland to assess whether IOL cases fulfilled the correct indication and methods of induction by comparing them to the guidelines. We observed an induction rate of 20.5%, which we consider to be average, given the fact that IOL is one of the most frequently performed obstetric procedures around the world, and a study by Marconi has shown that the induction rate in Europe ranges from 6.8% to 33% [34]. Our study has two main findings. First, adherence to the IOL guidelines in terms of the indication, methods, and monitoring of IOL was high. However, there is a room for improvement in the documentation process—especially the Bishop score. Second, multiparity and obesity were associated with the success of IOL.

### 4.1. Baseline Characteristics

Out of the 278 included deliveries, the mean age was 32 years, which is also the mean age in Switzerland and ranks quite high in Europe. This is an indication that women in Europe are having their children at a later stage in life, as shown in other studies and by the Swiss Federal Statistics Office [35,36]. The mean duration of induction in our study was 2.36 days, and 28.8% of the population were of advanced maternal age (AMA), which is associated with obstetric risk factors and neonatal complications [37]. Our study reported the median (IQR) GA as 40.4 (39.4–41.3) weeks, which indicates that most of the deliveries happened at full term. This corresponds to the study by Declercq et al., comparing the birth timing and GA in the United States to that of England and the Netherlands, which showed that more births happened during 39–40 weeks of gestation [38]. We also noted that most women were primipara; as it was their first childbirth, they were at a higher risk of experiencing prolonged labour and foetal distress than multiparous women [39]. The median LOS for vaginal delivery in our study was 5 days; for caesarean delivery, it was 6 days, which shows that women who gave birth through CS had a longer LOS. This does not correspond to the study by Hassan et al., which showed a shorter LOS for women who underwent CS (mean 2.7 days) [40]. However, other studies have shown and proposed a longer LOS in the case of CS compared to vaginal delivery [41,42].

### 4.2. Guideline Adherence

We aimed to assess the adherence of IOL in KSBL to the official guidelines. The majority of inductions were in line with the protocol based on the indication to carry out this procedure, although there was a little deviation from the guideline in a few cases. The pregnant women were educated and documentation of the procedure was recorded in all cases, although the Bishop score was not documented in 59% of the total population in the process. There was good adherence, especially in the use of multiple induction methods. With continuous CTG monitoring as a standard practice during labour to assess foetal well-being and detect any signs of foetal distress, we adhered fully to the guideline by monitoring and documenting throughout induction and childbirth; other studies have also shown that CTG monitoring plays a vital role in determining safer childbirth [43,44,45].

### 4.3. Factors Associated with Successful IOL

Our study did not find any association in most of the parameters when assessing successful IOL factors. However, when we explored the impact of parity on the success of induction, there was a statistically significant difference between primiparous and multiparous women (62.4% vs. 81.8% *p* = 0.001). Pregnancy-related risks, such as GBS, might influence this difference. This finding also suggests that primiparity is a potential risk factor for unsuccessful induction [46,47,48]. Notably, non-obese women appear to have a higher success rate for the induction of labour compared to obese women (73.4 vs. 54.1% *p* = 0.026). Therefore, it seems that body weight has an influence on the success of IOL. This finding corresponds with the study by Ellis et al. and showed that maternal obesity was associated with a prolonged birth duration and less frequent success of cervical ripening methods, and obese women were more likely to undergo a caesarean birth [49].

### 4.4. Maternal and Neonatal Outcomes

It was noted in our study that more than half of the pregnant women (54.7%) had at least a birth tear as a result of having a large foetus, foetal malpresentation, foetal distress, primiparity, or shoulder dystocia, with a high number of perineal (28.8%), vaginal (26.6%), and labia tears (15.8%). This does not correspond with a study by Jansson et al. that included 644 study samples, with almost half of the women (47.6%) having labia tears, (85.1%) vaginal tears, and (66.4%) perineal tears [50].

Most newborns had a good Apgar score of more than seven during the first minute, with an improvement in the fifth minute for newborns with an initial lower score. At the same time, 16.2% had moderate to severe acidosis, an indicator of foetal asphyxia (Table 5). A low Apgar score and low umbilical cord artery pH can be determinants for neonatal resuscitation [51,52]. As low birth weight, low Apgar score, and premature birth are outcomes that can lead to potential harm to neonatal health and possibly death [53], we recorded a low amount of these outcomes in our study, since 98% of the newborns were delivered alive, with a 4.3% resuscitation rate and a 0.7% mortality rate. This does not correspond to the study by Tavares et al., which recorded 79 newborns with a 5 min Apgar score <7 and 42 death cases [54].

### 4.5. Strengths and Limitations

Our study utilised real-life clinical routine data and aimed at an objective comparison to the guideline implementation, the inclusion of all IOL cases (except denial of consent and <18 years) in the representative population, and the inclusion of different maternal and neonatal outcomes to obtain a comprehensive picture. On the other hand, the study was conducted with a relatively small sample size of 278 participants; this size may restrict the generalizability of the findings to a larger population. Future studies with larger sample sizes may be needed to validate the results. Considering that our study was a single-centre study and retrospective, there is a possibility of incomplete or missing data, which could impact the accuracy of the results, while a multi-centre study involving a larger population could provide a more comprehensive understanding of the topic. Our study did not include a control group, making it challenging to directly compare with other groups, i.e., non-induced deliveries. The study also focused on specific variables; other potentially relevant variables such as stages of labour and the dose of each medication used that could influence the outcomes were not included, which may limit the comprehensive understanding of the topic.

## 5. Conclusions

In this retrospective study, we noted the high guideline adherence rate of IOL indications, methods of IOL, and the monitoring process. Nevertheless, improvements can be made in terms of the timing of induction and the use of oxytocin in the induction process. A major improvement is needed in the measurement and documentation of the Bishop score. We found that maternal age and GA did not significantly differ between successful and unsuccessful labour induction. At the same time, we found associations between parity and obesity with the success of labour induction.

Overall, this study contributes to the understanding of IOL practices in Switzerland by highlighting areas of adherence to guidelines and identifying factors that may influence the success of induction.

## Figures and Tables

**Table 1:** Patient-based baseline characteristics.

Baseline Characteristics	n = 278
Age (years), mean ± SD	32.18 ± 4.75
19–34, n (%)	198 (71.2)
≥35, n (%)	80 (28.8)
BMI (kg/m^2^), mean ± SD	29.83 ± 6.48
LOS (days) median (IQR)	5 (4–6)
Duration of induction, mean ± SD (days)	2.36 ± 1.58
Gravida	
GA (weeks), median (IQR)	40.4 (39.4–41.3)
Primigravida, n (%)	104 (37.4)
Multigravida, n (%)	171 (62.5)
Grandmultigravida, n (%)	3 (1.1)
Parity	
Primipara, n (%)	157 (56)
Multipara, n (%)	119 (43)
Grand multipara, n (%)	2 (1)
Pre-existing comorbidities, n (%)	77 (27.7)
Bronchial asthma, n (%)	12 (4.3)
Hypertension, n (%)	8 (2.9)
Endometriosis, n (%)	2 (0.7)
Hypothyroidism, n (%)	18 (6.5)
Iron deficiency anaemia, n (%)	31 (11.1)
Fibroid, n (%)	6 (2.2)

Abbreviations: BMI—body mass index (kg/m^2^); GA—gestational age; IQR—interquartile range; LOS—length of hospital stay; SD—standard deviation. Definition of grandmultigravida—5 or more pregnancies, grand multipara—5 or more deliveries beyond GA of 24 weeks.

**Table 2:** IOL according to the guideline indication.

Indications	Guideline	KSBL IOL According to Guideline
Missed deadline, n (%)	41+0–42+0	56 (20.1)
PROM, n (%)	37+0	46 (16.5)
GDM, n (%)	40+0	26 (9.3)
Oligohydramnios, n (%)	37+0	3 (1.1)
Polyhydramnios, n (%)	38+0 with additional risks	3 (1.1)
Intrahepatic cholestasis, n (%)	37+0	5 (1.8)
Preeclampsia, n (%)	≤37+0	9 (3.2)
HELLP syndrome, n (%)	34+0	0
Macrosomia, n (%)	39+0	9 (3.2)
IUGR/SGA, n (%)	26+0–38+0	9 (3.2)
Without medical reason, n (%)	≥39+0	3 (1.1)
Not documented, n (%)	-	4 (1.4)
Multiple indications, n (%)	Recommended	105 (38)

Abbreviations: GDM—gestational diabetes mellitus; HELLP—haemolysis, elevated liver enzymes, low platelet count; IOL—induction of labour; IUGR—intrauterine growth restriction; PROM—premature rupture of membrane; SGA—small for gestational age.

**Table 3:** Guideline implementation of the induction process and method of induction.

Other Recommendations	Guideline	KSBL IOL According to Guideline
Amniotomy alone, n (%)	Not recommended	0
Balloon catheter + Oxytocin, n (%)	Recommended	10 (3.6)
Balloon catheter + PGE1, n (%)	Recommended	0
Balloon catheter + PGE2, n (%)	Recommended	0
PGE2 alone, n (%)	Recommended alone	102 (36.7)
PGE1 + Oxytocin, n (%)	Recommended	24 (8.6)
PGE2 + PGE1, n (%)	Recommended	9 (3.2)
Use of multiple IOL methods, n (%)	Recommended	133 (47.8)
CTG monitoring, n (%)	Recommended	278 (100)
Education about the procedure, n (%)	Recommended	278 (100)

Abbreviations: CTG—cardiotocography; IOL—induction od labour; PGE1—prostaglandin E1; PGE2—prostaglandin E2; 0—no induction was carried out.

**Table 4:** Factors associated with successful induction.

Variables	Total	Successful IOL		*p*-Value
		Yes	No	
	278 (100)	197 (70.9)	81 (29.1)	
Maternal age				0.956
<35, n (%)	198 (71.2)	141 (71.2)	57 (28.8)	
≥35, n (%)	80 (28.8)	56 (70.0)	24 (30.0)	
Parity				0.001 *
Primiparous, n (%)	157 (56.5)	98 (62.4)	59 (37.6)	
Multiparous, n (%)	121 (43.5)	99 (81.8)	22 (18.2)	
Bishop score				0.645
Documented (unfavourable), n (%)	114 (41)	83 (72.8)	31 (27.2)	
Undocumented, n (%)	164 (59)	114 (69.5)	50 (30.5)	
GA (weeks)				0.511 ^±^
<37, n (%)	6 (2.2)	4 (66.7)	2 (33.3)	
37–41, n (%)	261 (93.9)	187 (71.6)	74 (28.4)	
≥42, n (%)	11 (4)	6 (54.5)	5 (45.5)	
Birth weight (g)				0.839 ^±^
<2500, n (%)	12 (4.3)	8 (66.7)	4 (33.3)	
2500–4000, n (%)	217 (78.1)	153 (70.5)	64 (29.5)	
>4000, n (%)	49 (17.6)	36 (73.5)	13 (26.5)	
Risks				
No GBS, n (%)	239 (86)	173 (72.4)	66 (27.6)	0.233
GBS, n (%)	39 (14)	24 (61.5)	15 (38.5)	
Non-obese, n (%)	241 (86.7)	177 (73.4)	64 (26.6)	0.026 *
Obesity, n (%)	37 (13.3)	20 (54.1)	17 (45.9)	
No preeclampsia, n (%)	260 (93.5)	187 (71.9)	73 (28.1)	0.226
Preeclampsia, n (%)	18 (6.5)	10 (55.6)	8 (44.4)	
NO PROM, n (%)	219 (78.8)	154 (70.3)	65 (29.7)	0.824
PROM, n (%)	59 (21.2)	43 (72.9)	16 (27.1)	
No GDM, n (%)	226 (81.3)	157 (69.5)	69 (30.5)	0.37
GDM, n (%)	52 (18.7)	40 (76.9)	12 (23.1)	
No IUGR, n (%)	254 (91.4)	179 (70.5)	75 (29.5)	0.817
IUGR, n (%)	24 (8.6)	18 (75.0)	6 (25.0)	

GA—gestational age; GBS—group B streptococcus; GDM—gestational diabetes mellitus; IUGR—intrauterine growth restriction; PROM—premature rupture of membrane; *p*-values < 0.05 were considered statistically significant. A chi-squared test for significance testing was used for the analyses. Significance codes: * <0.05, ^±^ Fisher’s exact test was used for significance testing.

**Table 5:** Maternal and neonatal well-being in induced labour.

Outcomes of Induced Deliveries	
Mode of delivery	
Vaginal, n (%)	197 (70.9)
Caesarean section, n (%)	81 (29.1)
Birth injuries, n (%)	152 (54.7)
Episiotomy, n (%)	31 (11.2)
Vaginal tear, n (%)	74 (26.6)
Perineal tear, n (%)	80 (28.8)
Labia tear, n (%)	44 (15.8)
Paraurethral tear, n (%)	2 (0.7)
Cervical tear, n (%)	1 (0.4)
Postpartum haemorrhage	266 (95.7) ^a^
≤500 mL, n (%)	184 (66.2)
>500 mL, n (%)	58 (20.9)
≥1000 mL, n (%)	24 (8.6)
Infections	25 (9)
Amniotic infection syndrome, n (%)	13 (4.6)
Genital warts, n (%)	1 (0.4)
Candidiasis, n (%)	2 (0.7)
Chlamydia, n (%)	1 (0.4)
Herpes, n (%)	1 (0.4)
Active HBV, HCV, HEV, n (%)	7 (2.5)
Apgar at 1 min	
≤3, n (%)	13 (4.7)
4–6, n (%)	16 (5.8)
≥7, n (%)	249 (89.5)
Apgar at 5 min	
≤3, n (%)	3 (1.1)
4–6, n (%)	7 (2.5)
≥7, n (%)	268 (96.4)
Umbilical cord artery pH	215 (77.3) ^b^
7.20–7.40, n (%)	168 (60.4)
<7.20, n (%)	45 (16.2)
>7.40, n (%)	2 (0.7)
Resuscitation, n (%)	12 (4.3)
Livebirth, n (%)	272 (98) ^c^
Abortion induction (14–26 GW), n (%)	2 (0.7)
Birth weight	
SGA (1500–2500), n (%)	12 (4.3)
Normal birth weight (2500–4000), n (%)	217 (78.1)
LGA (>4000), n (%)	49 (17.6)

Missing data ^a^—12 (4.3%) ^b^—63 (22.6%) ^c^—4 (1.4%) GW—weeks of gestation, HBV—hepatitis b virus; HCV—hepatitis c virus; HEV—hepatitis e virus; LGA—large for gestational age; SGA—small for gestational age.

## Data Availability

The data presented in this study are available on request from the corresponding author. The data are not publicly available due to data privacy restrictions.
